# Fungicidal activity of peptides encoded by immunoglobulin genes

**DOI:** 10.1038/s41598-017-11396-6

**Published:** 2017-09-07

**Authors:** Luciano Polonelli, Tecla Ciociola, Martina Sperindè, Laura Giovati, Tiziana D’Adda, Serena Galati, Luiz R. Travassos, Walter Magliani, Stefania Conti

**Affiliations:** 1 Department of Medicine and Surgery, University of Parma, Parma, Italy; 2 Department of Chemistry, Life Sciences and Environmental Sustainability, University of Parma, Parma, Italy; 3 Experimental Oncology Unit, Federal University of São Paulo (UNIFESP), São Paulo, Brazil

## Abstract

Evidence from previous works disclosed the antimicrobial, antiviral, anti-tumour and/or immunomodulatory activity exerted, through different mechanisms of action, by peptides expressed in the complementarity-determining regions or even in the constant region of antibodies, independently from their specificity and isotype. Presently, we report the selection, from available databases, of peptide sequences encoded by immunoglobulin genes for the evaluation of their potential biological activities. Synthetic peptides representing the translated products of *J* lambda and *J* heavy genes proved to act *in vitro* against pathogenic fungi, entering yeast cells and causing their death, and exerted a therapeutic effect in a *Galleria mellonella* model of infection by *Candida albicans*. No haemolytic, cytotoxic and genotoxic effects were observed on mammalian cells. These findings raise the hypothesis that antibodies could be the evolutionary result of the adaptive combination of gene products ancestrally devoted to innate antimicrobial immunity.

## Introduction

It is well known that the entire repertoire of human immunoglobulins (Igs) comprises such a vast number of different molecules to virtually recognise and bind to, through the antibody (Ab) variable region, any non-self structure, and even self-epitopes, as is the case of auto-Abs. In contrast, the constant region of Abs is mainly involved in the effector functions and has less variability. Although several different genes (*V* and *J* for light chains, *V*, *D* and *J* for heavy chains) encode the variable Ig domains, they are certainly not enough to justify the large number of different Igs in nature. Differentiated plasma cells produce diverse Abs through the *V*(*D*)*J* recombination, a gene rearrangement process in which the introns are removed, and the various sequences form pairs of different light and heavy variable regions^1, 2^. Moreover, junctional diversity results by addition or subtraction of nucleotides during the recombination process between *VJ* and *VDJ* genes. Further diversity derives from the somatic hypermutation mechanism, which occurs in the *V* gene after recombination^3^.

In previous studies, we have shown that synthetic peptides with sequences identical to Ig fragments related to complementarity-determining regions (CDRs) and to constant domains can exert *in vitro*, *ex vivo* and/or *in vivo* antimicrobial, antiviral, immunomodulatory and/or anti-tumour activities, regardless of the specificity and isotype of the belonging Ab^4–8^. More recently, a naturally occurring IgM fragment proved to exert antifungal and antiviral activity, and also showed a therapeutic effect against an experimental *Candida albicans* infection^9^.

These findings led to the speculation that the products of Ig genes could be endowed with innate anti-infective activity. Synthetic peptides with the sequence of selected *J* lambda and *J* heavy gene products proved to display a fungicidal activity *in vitro* and a therapeutic activity in *Galleria mellonella* larvae against systemic candidiasis.

These results support the hypothesis that genes encoding peptides ancestrally devoted to innate immunity may have been combined in the course of evolution to give rise to antibody molecules, characterised by highly specific activity, as effectors of adaptive immunity.

## Results

### Selection of peptides encoded by immunoglobulin genes

The search for peptides encoded by immunoglobulin genes (loci *lambda*, *kappa* and *heavy*) resulted in the selection of four peptides, denominated L12P, W12K, G10S and L18R. The amino acid sequences and characteristics of the selected peptides are shown in Table 1. The selected peptides were synthesised and evaluated for *in vitro* fungicidal activity against reference yeast strains.

**Table 1:** Characteristics of the selected peptides encoded by immunoglobulin genes.

Peptide	Locus	Gene	Amino acid sequence	Hydrophobicity^a^	pI	M.M.	Net charge
L12P	Lambda	IGLJ1	LCLRNWDQGHRP	0*0+*0-*0++0	8.26	1494.7	2+
W12K	Kappa	IGKJ1	WTFGQGTKVEIK	0*00*0*+0-0+	8.59	1393.7	+
G10S	Heavy	IGHD2-15	GYCSGGSCYS	00**00**0*	5.51	983.2	0
L18R	Heavy	IGHJ2	LLVLRSLGPWHPGHCLLR	0000+*0000+00+*00+	10.35	2068.1	4+

^a^0: hydrophobic, *polar, +, and −: positively charged and negatively charged residues; pI: isoelectric point; M.M.: molecular mass (Daltons).

### *In vitro* biological activity of the selected peptides

#### Fungicidal activity

The selected peptides L12P and L18R exhibited a significant activity against all the investigated yeast strains (representative images in Supplementary Fig. S1), with half maximal effective concentration (EC_50_) values ranging from 0.188 μM (L18R against *Cryptococcus neoformans* 6995) to 0.658 μM (L12P against *Malassezia furfur* 101) (Table 2). The peptides W12K and G10S showed a weaker activity, if any. In preliminary assays performed at the concentration of 100 μg/ml, their inhibitory activity ranged from 0% to 89% in comparison to the control growth (in the absence of peptides), with the only exception of peptide W12K against *C. neoformans* 6995 (100% inhibition at 100 μg/ml, EC_50_ 12.91 μM).

**Table 2:** *In vitro* fungicidal activity of the selected peptides L12P and L18R.

Yeast strain	EC_50_* (95% confidence intervals) [mol/liter] × 10^−6^	
	L12P	L18R
*Candida albicans* SC5314	0.489 (0.445–0.538)	0.443 (0.437–0.549)
*C. albicans* CA-6	0.556 (0.522–0.591)	0.294 (0.292–0.296)
*C. albicans* SA40	0.537 (0.531–0.544)	0.315 (0.271–0.366)
*C. albicans* AIDS68	0.501 (0.468–0.537)	0.449 (0.435–0.463)
*C. albicans* UM4	0.627 (0.534–0.736)	0.454 (0.414–0.499)
*C. glabrata* OMNI32	0.546 (0.520–0.574)	0.356 (0.339–0.373)
*Cryptococcus neoformans* 6995	0.364 (0.353–0.375)	0.188 (0.180–0.196)
*Malassezia furfur* 101	0.658 (0.574–0.754)	0.527 (0.472–0.586)

*EC_50_, half maximal effective concentration, calculated by nonlinear regression analysis using Graph Pad Prism 4.01 software.

Time-killing curves, determined by incubation over time of *C. albicans* SC5314 cells with L12P and L18R at their minimal fungicidal concentration (5 μg/ml), demonstrated a very rapid candidacidal effect of the peptides. In particular, nearly 93% and 95% killing with L12P and nearly 97% and more than 99% killing with L18R was observed in 5 and 10 min, respectively (Supplementary Fig. S2).

#### Haemolytic, cytotoxic, and genotoxic effects

Haemolytic, cytotoxic and genotoxic effects were evaluated on human erythrocytes, mammalian cells and peripheral blood mononuclear cells (PBMCs), respectively. The selected peptides did not show a significant haemolytic activity, either after 30 or 120 min of incubation. Even at the highest tested concentration (500 μM) less than 1% of the erythrocytes lysed with reference to the negative control (0% lysis), consisting of erythrocytes suspended in phosphate buffered saline (PBS), in comparison to the positive control (erythrocytes suspended in PBS plus Triton 1%, 100% lysis). As the only exception, higher values of haemolysis, although always less than 10%, were observed after 120 min in the presence of increasing concentrations of L18R (4.4% at 50 μM, 5.8% at 100 μM, 7.5% at 250 μM and 9.6% at 500 μM) (Supplemetary Table S1).

None of the selected peptides was significantly cytotoxic against LLC-MK2 cells, as assessed by a cell viability assay using resazurin as indicator. At all concentrations tested, mean values of fluorescence intensity did not differ significantly from the ones of the untreated cells, with the exception of L18R at the highest concentrations. In the presence of this peptide cell viability was reduced, in comparison to untreated control (100% viability), to 90.14% and 83.88% at 250 and 500 μM respectively (concentrations several hundred times higher than the EC_50_) (Supplemetary Table S2).

No genotoxic activity was detected in the Comet assay performed on PBMCs with the most active peptides (Supplemetary Table S3). Tail DNA values were always very low (<1%) for PBMCs treated with 5 and 10 μM L12P and L18R (0.55 ± 0.08, 0.21 ± 0.14, 0.22 ± 0.02 and 0.21 ± 0.06 respectively, in comparison with the negative control value of 0.37 ± 0.04 recorded for untreated PBMCs). Accordingly, there were no significant changes in the visual score values (109 ± 4.24, 103 ± 1.41, 106 ± 1.00, and 103 ± 1.41, in the presence of 5 and 10 μM L12P and L18R respectively, in comparison with 104 ± 2.83 in the absence of peptides).

### *In vivo* toxicity and therapeutic activity of peptides

**Figure 1:**
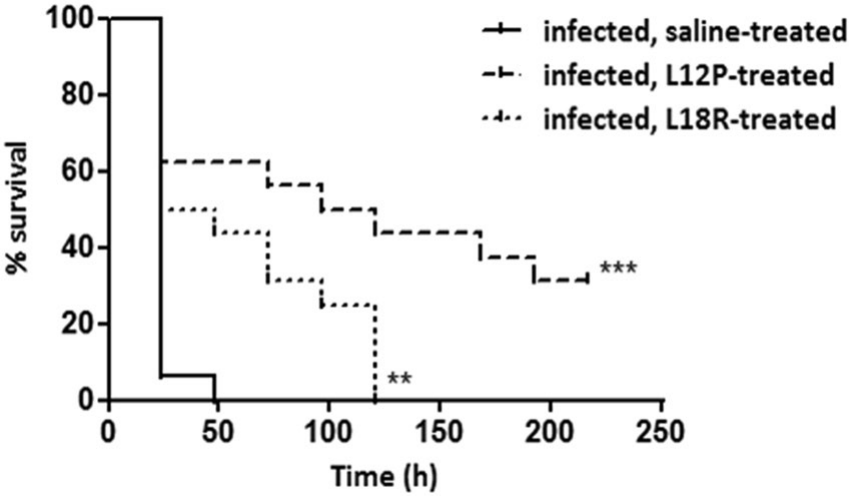
*In vivo* therapeutic activity of L12P and L18R peptides. Groups of 16 *Galleria mellonella* larvae were infected with 5 × 10^5^ cells of *Candida albicans* SC5314 and administered 1 h later (single injection, 10 μl) with peptides (12.5 mg/kg) or saline solution (control group). Larvae were then incubated at 37 °C in the dark for 9 days, and scored daily for survival.The survival curves of peptide-treated animals were significantly different from that of control group, as assessed by the log-rank (Mantel-Cox) test (**p = 0.0016, ***p = 0.0001). Data reported are from one representative experiment out of two experiments with comparable results.

Toxicity to the host and therapeutic activity against *C. albicans* infection were evaluated for L12P and L18R peptides in *G. mellonella* larvae. No significant difference in survival was detected between larvae inoculated with saline (control group) or with the peptides (12.5 mg/kg) showing the lack of toxicity under the adopted experimental conditions. In two independent experiments, a single administration of both peptides (12.5 mg/kg) led to a significant increase in survival of larvae infected with *C. albicans* cells in comparison to that of control group, i.e. infected larvae inoculated with saline. The survival curves of one representative experiment, showing a significant statistical difference (*p* = 0.0001 and *p* < 0.002 for L12P and L18R, respectively) are depicted in Fig. 1. Median survival time was 108 h in L12P-treated group and 36 h in L18R-treated group vs 24 h in saline-injected control group. While 100% of the untreated larvae were dead 48 h post-infection, survival was prolonged up to 120 h in the L18R-treated group, while 5/16 of the L12P-treated larvae were still alive at day 9.

### Induction of apoptosis in *C. albicans* cells

**Figure 2:**
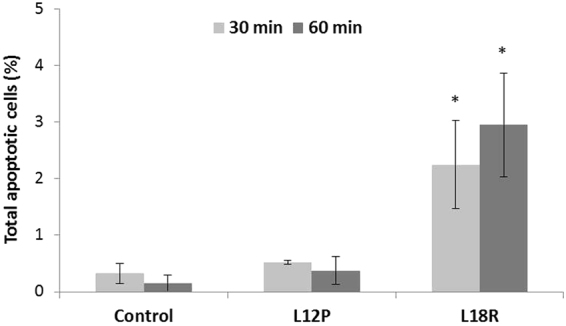
Apoptotic effects of the L12P and L18R peptides on *Candida albicans* SC5314 cells. Phosphatidylserine externalisation was analysed by flow cytometry after 30 and 60 min of treatment with peptides at 2 × EC_50_. While no difference was observed between untreated cells (control) and cells treated with L12P, after treatment with L18R a significant, although low, percentage of apoptotic cells was observed. Data represent the means ± standard deviations from two independent experiments (*p < 0.05 vs control untreated cells).

Phosphatidylserine externalisation and reactivity with annexin V was determined by flow cytometry and used to assess whether apoptosis is induced in *C. albicans* SC5314 whole cells after 30 and 60 min treatment with the L12P and L18R peptides at 2 × EC_50_. Under the adopted experimental conditions, the percentage of apoptotic cells on the total gated cells was not significantly different in L12P-treated cells in comparison with untreated control cells. Conversely, a significant, although low, percentage of apoptotic cells was observed after treatment with L18R peptide (Fig. 2). A CFU assay performed on peptide-treated in comparison to control untreated cells confirmed the candidacidal activity of the peptides under the adopted experimental conditions.

### Peptide-yeast cell interaction

**Figure 3:**
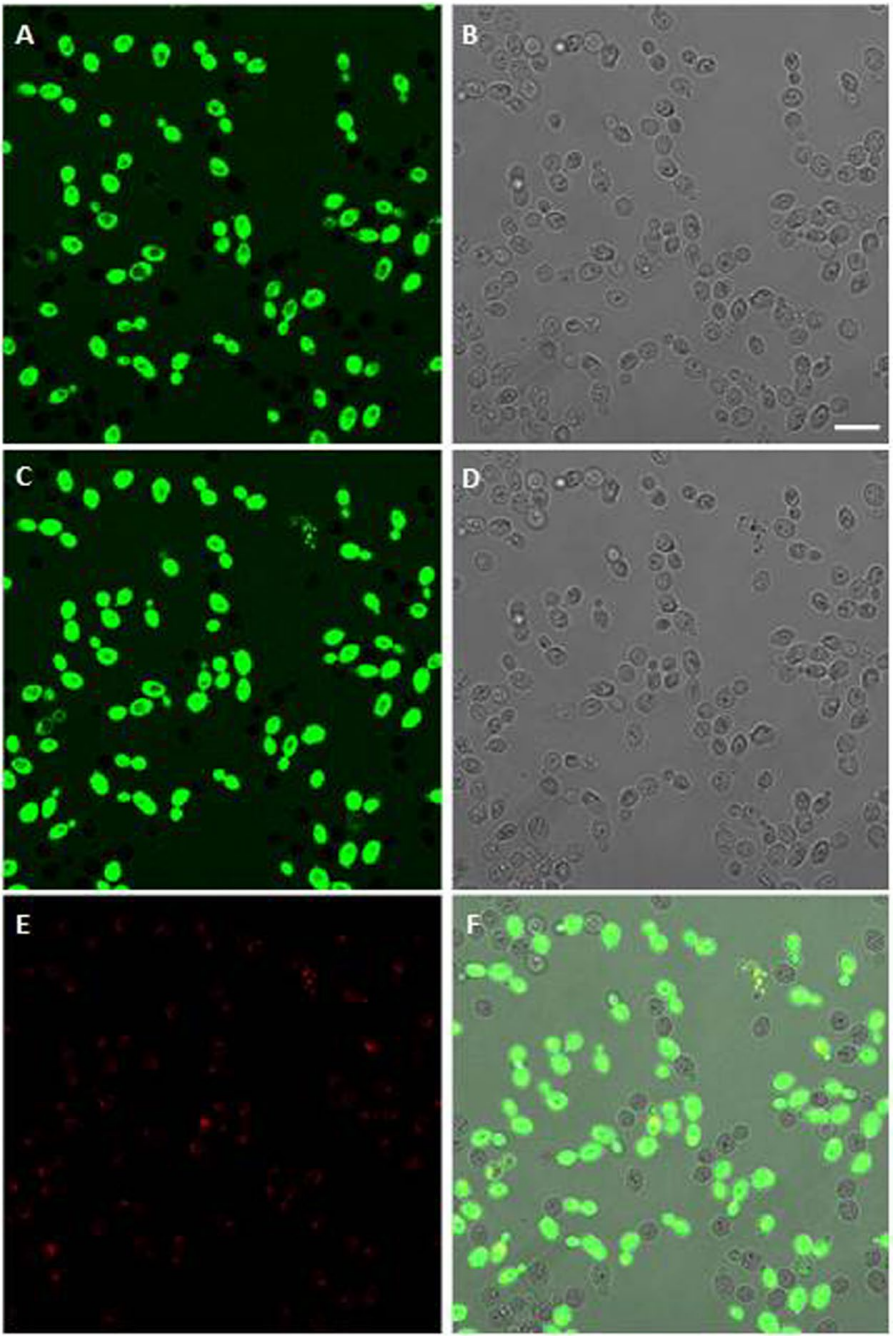
Internalisation of the L12P peptide into *Candida albicans* SC5314 cells. Confocal images of living yeast cells incubated in the presence of the fluorescein-labeled peptide for 5 min and 15 min are presented in panels A and C, respectively. The same field is shown by light transmission images in panels B (5 min) and D (15 min). L12P entered into most yeast cells within few minutes; empty vacuoles were seen. After 15 min, some yeast cells were entirely fluorescent and no longer viable as assessed by propidium iodide internalisation (panel E). Panel F: merge of panels C and E. Bar = 10 μm.

**Figure 4:**
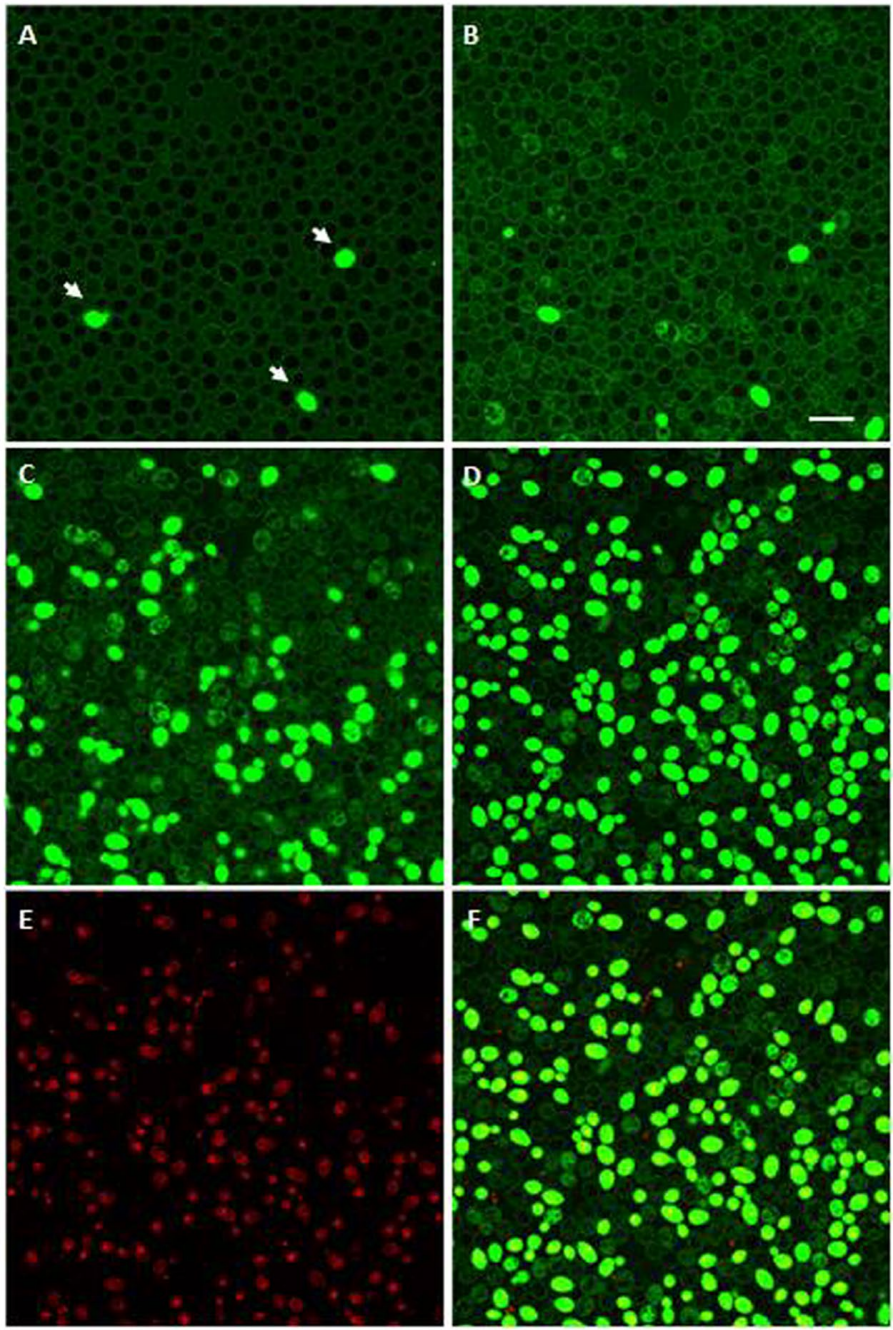
Internalisation of the L18R peptide into *Candida albicans* SC5314 cells. Confocal images of living yeast cells incubated in the presence of the fluorescein-labeled peptide for 5 min, 30 min, 50 min and 65 min are presented in panels A to D. The same field is shown. Labeled L18R bound to the surface of all yeast cells in a few minutes, then was progressively internalised by yeast cells. After 65 min, fluorescence was diffused in yeast cells that were no longer viable as assessed by propidium iodide internalisation (panel E). Panel F: merge of panels C and E. Non-viable yeast cells in the inoculum passively internalised L18R peptide (panel A, arrows). Bar = 10 μm.

Time-lapse confocal microscopy allowed the investigation of the interaction between fluorescein isothiocyanate (FITC)-labeled L12P and L8A peptides and living *C. albicans* SC5314 cells. Both peptides entered yeast cells, gathered inside and led to cell death, but in different patterns. As shown in Fig. 3, after 5 min the FITC-labeled L12P peptide was found inside most *C. albicans* cells, except within vacuoles. After 15 min, some cells were completely fluorescent and already dead, as shown by propidium iodide internalisation. Conversely, after 5 min peptide L18R bound to the surface of all yeast cells (Fig. 4, panel A), and progressive internalisation and compartmentalisation were observed over time (Fig. 4, panels B,C). A diffuse fluorescence was observed only in dead cells (Fig. 4, panels D–F).

### Visualisation of the effects of L12P and L18R peptides on *C. albicans* cells by transmission and scanning electron microscopy

**Figure 5:**
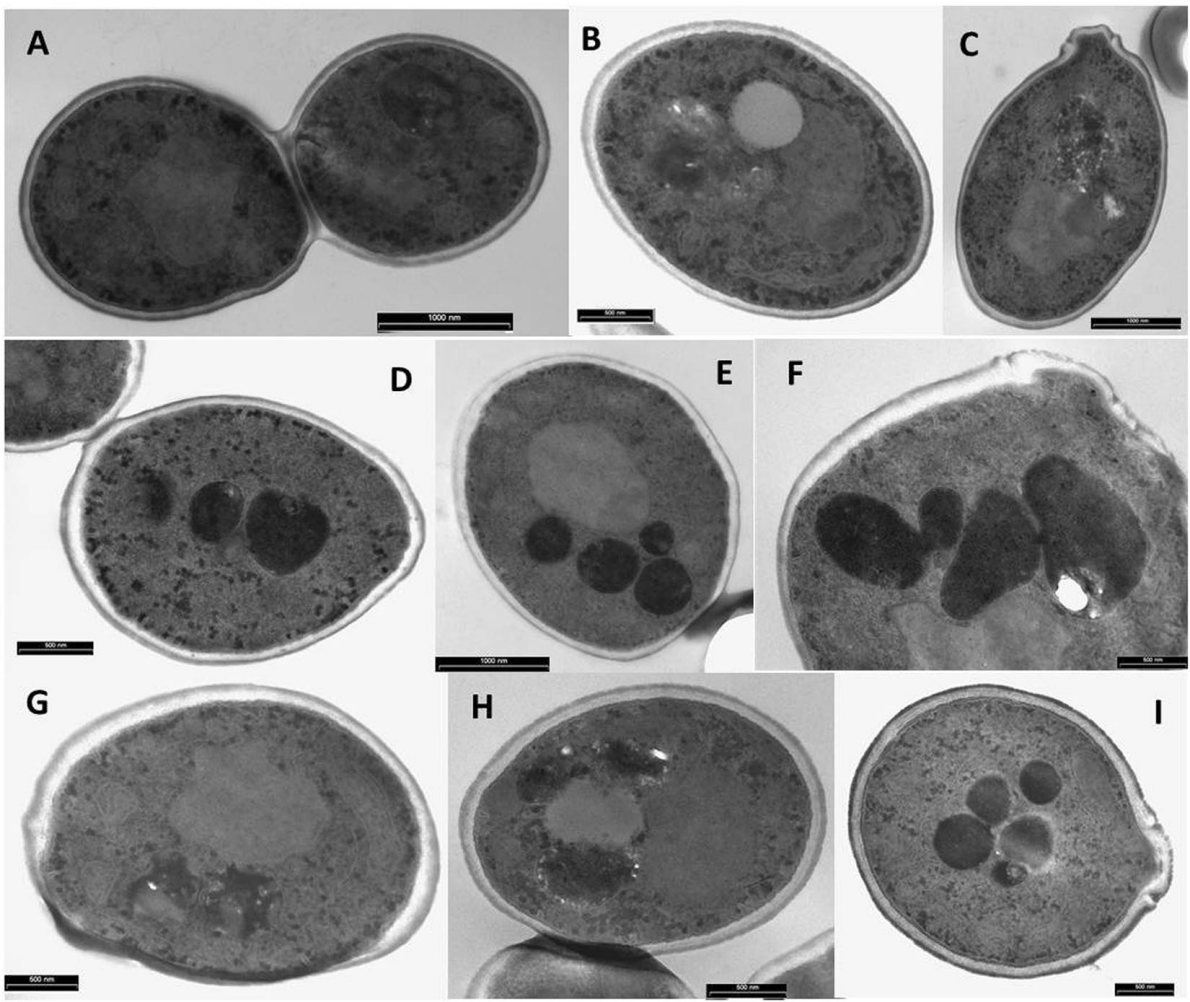
Transmission electron microscopy of *Candida albicans* cells treated with L12P and L18R peptides. Approximately 10^7^ yeast cells were incubated without (panels A–C, controls) or with the selected peptides (125 μg/ml) (panels D–F, cells treated with peptide L12P; panels G–I, cells treated with peptide L18R) for 60 min. Still intact cells treated with both peptides presented cytoplasmic changes with the presence of a number of microbodies (panels D,E,F,I), membrane retraction and cell wall alteration (panels D,F,G,H). Bar: 1000 nm (panels A,C,E), 500 nm (panels B,D,F–I).

**Figure 6:**
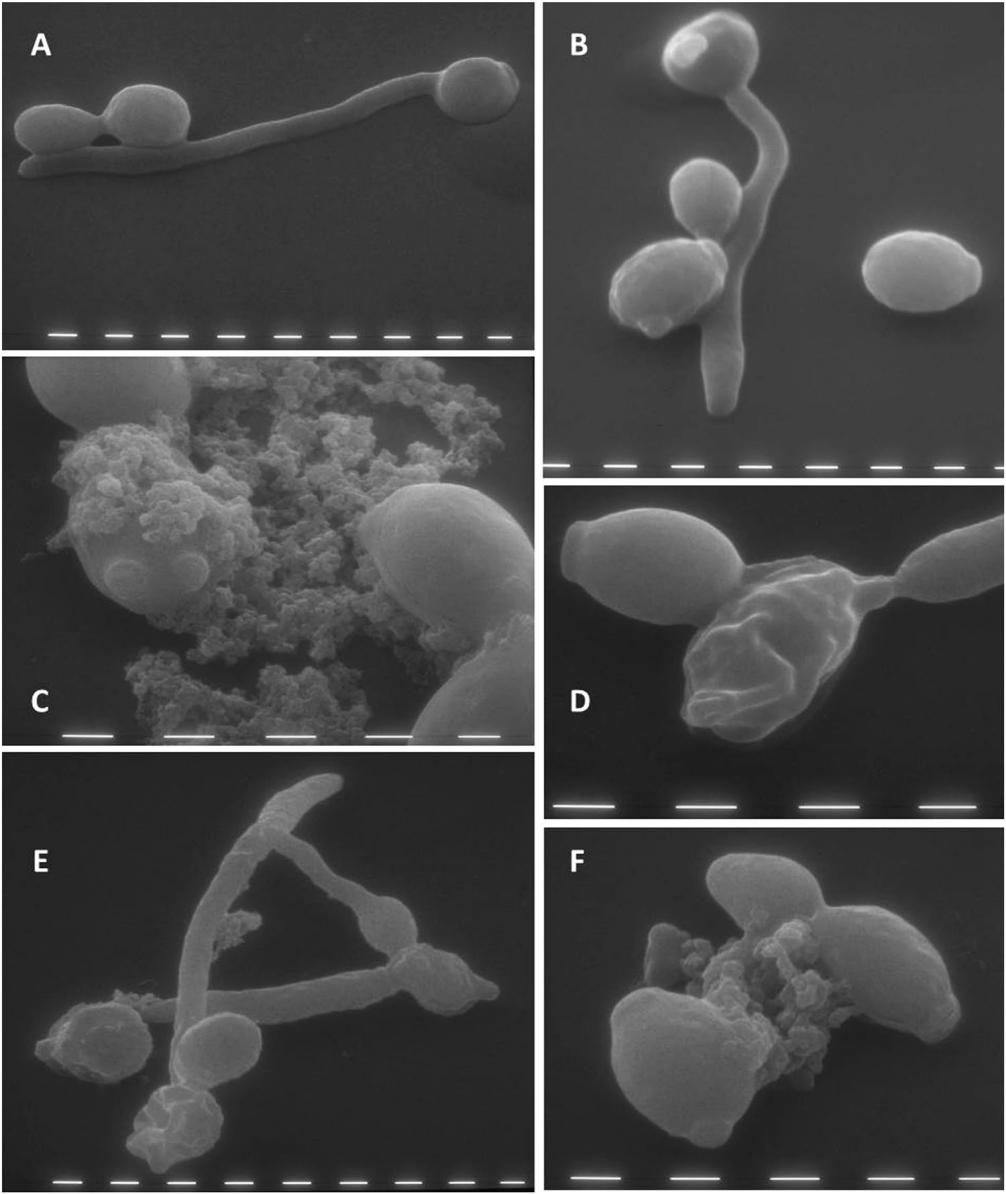
Scanning electron microscopy of *Candida albicans* cells treated with L12P and L18R peptides. Approximately 4 × 10^5^ yeast cells were incubated without (panels A and B, controls) or with the selected peptides (125 μg/ml) (panels C and D, cells treated with peptide L12P; panels E and F, cells treated with peptide L18R) for 60 min. Masses of debris and cell leakage (panels C, E, F), and gross alterations in cell surface (panels D and E) were observed after treatment with both peptides. Bar: 1 μm.

As shown in Figs 5 and 6, treatment with L12P and L18R peptides caused alterations in the morphology of *C. albicans* cells in comparison to untreated controls. In transmission electron microscopy specimens, microbodies were seen in still intact treated cells. Cell surface alterations were also observed. Scanning electron microscopy showed masses of cellular debris, apparent cell leakage and deformed yeast cells.

## Discussion

Over the years, hundreds of peptides with antimicrobial, antiviral and/or immunomodulatory activity have been isolated and characterised from diverse vertebrate and invertebrate animals, plants and eukaryotic and prokaryotic microorganisms. More than 2,800 of such molecules are currently listed in the Antimicrobial Peptide Database (APD3)^10^. Most antimicrobial peptides, also referred to as host defence peptides, share common features, such as small size, net positive charge, an amphipathic structure and affinity for negatively charged phospholipids^11–15^. In humans, diverse gene-encoded cationic peptides, as α-defensins, cathelicidin LL-37, histatins and hepcidin, are produced and play a role in host defense^11, 13, 15, 16^. Besides these natural molecules recognised to be involved in innate immunity, bioactive peptides may derive from the proteolytic cleavage of physiological proteins. Haemoglobin is the source of antimicrobial peptides^17, 18^, including HB33–61 from the α-chain of bovine haemoglobin, active at micromolar concentrations against Gram-positive bacteria and fungi^19^. Additional examples are buforins, derived from the protein histone H2A^20^, the 7–42 fragment from gastric inhibitory polypeptide^21^, lactoferricins from lactoferrin^22^, fragments of lactalbumin^23^, and derivatives from the precursor molecule pro-opiomelanocortin such as α-melanocyte-stimulating hormone and its carboxy-terminal tripeptide (11–13, KPV), able to inhibit *Staphylococcus aureus*, *C. albicans* and HIV-1 at picomolar concentrations^24, 25^. Short peptides derived from CDR H3 sequences proved to maintain the binding properties of the parental Ab and also to exert antiviral^26, 27^ and even anti-tumour activities^28^.

In our previous works, we demonstrated that synthetic peptides related to CDRs and domains of the constant regions of Abs of different classes may display antimicrobial, antiviral, immunomodulatory and/or anti-tumour activities, mediated by different mechanisms of action, entirely unrelated to the specificity of the originating Ab^4–8^.

The aim of this work was to evaluate, as a proof of concept, the potential microbicidal activity of synthetic peptides (<20 residues), representing the translated products of *J* and *D* genes selected from available databases.

The selected peptides showed a direct fungicidal activity, at micromolar concentrations, against several yeast species, including *Candida* strains resistant to conventional antifungal drugs. None of the investigated peptides showed haemolytic, cytotoxic or genotoxic activity *in vitro* against mammalian cells. The absence of *in vivo* toxicity in the *Galleria* model was also verified.

L12P and L18R were the most active peptides. Both proved to kill cells of *C.* *albicans* in a few minutes. Following treatment of *C. albicans* cells with L18R peptide a significant apoptotic cell death was detected.

The yeast cell penetration mechanism of L12P could likely depend on the cationic R residues that can interact with membrane phospholipids in a way Trojan peptides do. Several molecules of L12P would be sufficient to cause local disruption of the lipid core. As to peptide L18R, an interaction with the membrane seems plausible considering its hydrophobic face. Direct penetration via an energy independent pathway may involve stable or transient destabilisation of the membrane, associated with peptide folding on the lipid membrane. Subsequent internalisation depends on the peptide concentration, sequence and lipid composition of the membrane.

The main alterations observed by transmission electron microscopy in C. *albicans* cells treated with L12P and L18R (Fig. 5) are the presence of microbodies (peroxisomes) and the retraction of the plasma membrane coupled with cell wall alterations. It is conceivable that peptide action on the plasma membrane may generate precursors that have a role in microbodies formation. Microbodies may contain catalase and other enzymes, such as flavin-dependent alcohol oxidase and D-amino acid oxidase, and in the case of apoptosis oxidative molecules may include ROS, thioredoxin and other species. Membrane retraction and cell wall alterations probably reflect peptide effects on the cytoskeleton, actin filaments and other structures, that may lead to mitochondrial dysfunction.

Observations by scanning electron microscopy (Fig. 6) revealed deformed yeast cells and leakage of cellular components. Some cells appeared with a rugged surface suggesting alterations on the cell wall.

The therapeutic activity of L12P and L18R has been demonstrated in an experimental model of systemic infection by *C. albicans* in larvae of *G. mellonella*, a non-mammalian system which allows a reliable evaluation of *in vivo* efficacy of *in vitro* active molecules indipendently from adaptive immunity^29^. A significant increase in the survival of infected larvae was observed after a single peptide injection.

Overall, peptides encoded by Ig genes exerted an effective antifungal activity. Other possible biological functions (antibacterial, antiviral, anti-tumour, immunoregulatory), already recognised for previously described Ab-derived peptides^4, 5, 7, 8^, could be investigated in future studies.

These findings raise the hypothesis that ancestral genes, encoding peptides with nonspecific antimicrobial activity, could have been associated, in the course of evolution, to give rise to complex antibody molecules displaying highly specific activities. Such theory, along with the finding, in the human serum, of a peptide undoubtedly derived from IgM constant region able to exert diverse anti-infective activities^9^, may help to establish an unsuspected link between innate and adaptive immunity.

## Methods

### Ethics statement

Blood components were collected from periodical donors of the Transfusion Unit of the Azienda Ospedaliera Universitaria di Parma according to the policy of the Italian National Blood Centre Guidelines (April 5, 2013). Research did not involve interaction with the donors nor their identification.

### Selection and synthesis of peptides encoded by immunoglobulin genes

The research focused on *J* gene segments (lambda and kappa loci) for the light chain and *J* and *D* gene segments (heavy locus) for the heavy chain, exploiting the Gene database of the National Center for Biotechnology Information (NCBI, https://www.ncbi.nlm.nih.gov). Translated products were selected according to different criteria, i.e. presence of positively charged residues, net charge, isoelectric point, and alternation of hydrophobic/hydrophilic residues in the sequence, by using ExPASy Proteomics Tools Compute pI/MW and ProtParam (http://www.expasy.org/proteomics). Selected peptides were synthesised as previously described^9^ by solid phase peptide synthesis method using a multiple peptide synthesiser (SyroII, MultiSynTech GmbH), at CRIBI Biotechnology Center (University of Padua, Italy). The purity of peptides, evaluated by analytical reverse phase HPLC, was in the 80–90% range. The peptides were solubilised in dimethylsulfoxide at a concentration of 20 mg/ml and subsequently diluted in sterile distilled water for experimental use. For all experiments, controls (in the absence of peptides) contained dimethylsulfoxide at the proper concentration.

### Evaluation of the *in vitro* fungicidal activity of the selected peptides

The fungicidal activity of the selected synthetic peptides was evaluated *in vitro* by a previously described colony forming unit (CFU) assay against five strains of *C. albicans* (SC5314, CA-6, SA40, AIDS68, and UM4), *C. glabrata* OMNI32, *C. neoformans* serotype A 6995, and *Malassezia furfur* 101^6^. Briefly, approximately 500 viable yeast cells were incubated for 6 h at 37 °C in the absence (control) or presence of the selected peptides at scalar concentrations. The yeast suspensions were then seeded on plates of Sabouraud dextrose agar (SDA) (*Candida* spp. and *C. neoformans*), or SDA added with 1% Tween 20 (*M. furfur*), and colonies were enumerated after 48–72 h of incubation at 30 °C. Percent killing was calculated with reference to the number of colonies in controls. Each assay was performed in triplicate. The EC_50_ was calculated by nonlinear regression analysis using Graph Pad Prism 4.01 software, San Diego, CA, USA. Time kinetics of killing of *C. albicans* SC5314 was determined, for the most active peptides, by CFU assays after incubation of yeast cells for 5, 10, 20, 30, 60, 120, 240, and 360 min with 5 μM peptides.

### Evaluation of the haemolytic, cytotoxic, and genotoxic activity of the selected peptides

The synthetic selected peptides were evaluated for haemolytic activity against human erythrocytes (blood group 0 Rh+ ) according to a previously described procedure^6^. Briefly, 50, 100, 250 e 500 μM peptides in PBS were added to erythrocyte suspensions (final erythrocyte concentration, 2.5% v/v). Release of haemoglobin was monitored, at 30 and 120 min incubation at 37 °C, by measuring the absorbance at 540 nm of the supernatant obtained after centrifugation at 800 *g* for 10 min. Results were expressed as the percentage of lysed erythrocytes, where controls for zero haemolysis (blank) and 100% haemolysis consisted of erythrocytes suspended in PBS and 1% Triton X-100, respectively.

Cytotoxicity against mammalian cells was tested exploiting the ability of metabolically active cells to convert resazurin to fluorescent resorufin, as previously described^9^, using the cell line LLC-MK2, already available at our laboratory. Briefly, LLC-MK2 monkey kidney epithelial cells cultured on 96-well plates in Eagle’s Minimum Essential Medium with 2% fetal bovine serum were treated with 50, 100, 250 e 500 μM peptides for 24 h. Cells in medium without peptide served as control. After this period, cells were incubated with resazurin 44 μM in serum-free medium for 30 min at 37 °C, then fluorescence intensity was measured at 572 nm. Cell viability was expressed as the percent ratio T/C, where T represents the mean value obtained for cells treated with the peptides and C the mean value of control.

Genotoxic activity against human PBMCs was evaluated by alkaline Comet assay, as previously described^6^. Briefly, PBMCs cultured in RPMI 1640 medium were treated for 120 min with 5 and 10 μM L12P and L18R at 37 °C in an atmosphere containing 5% CO_2_. Cells were exposed at 4 °C overnight to a lysis buffer (2.5 M NaCl, 10 mM Na_2_EDTA, 10 mM Tris-HCl, 1% Triton X-100 and 10% dimethylsulfoxide, pH 10), then DNA unwinding was achieved over 20 min in an electrophoretic alkaline buffer (1 mM Na_2_EDTA, 300 mM NaOH, at 0 °C, pH >13). The electrophoresis was carried out for 20 min (0.78 V/cm, 300 mA) at 0 °C in the same buffer, followed by neutralisation in 0.4 M Tris-HCl, pH 7.5. The slides, stained with 0.75 μl ethidium bromide (10 μg/ml) were examined with a fluorescent microscope (Leica DMLS), equipped with a BP 515–560 nm excitation filter and an LP 580 nm barrier filter and data were collected using an automatic image analysis system (Comet Assay III, Perceptive Instruments Ltd). Fifty randomly-selected cells per slide (two slides per sample) were analysed. DNA migration was evaluated by percentage of DNA in comet tail (mean Tail Intensity). A visual score was also calculated after direct observation by the operator. The length of the comet tail allowed the attribution of the observed cells to a class (0 to 4) and the following formula was used: Visual score = number (no.) of class 0 cells + 2 × (no. of class 1 cells) + 3 × (no. of class 2 cells) + 4 × (no. of class 3 cells) + 5 × (no. of class 4 cells).

### Evaluation of *in vivo* toxicity and therapeutic activity of selected peptides

*In vivo* toxicity and potential therapeutic efficacy of selected peptides were studied in the *G. mellonella* model, as previously described^30^. To evaluate peptide toxicity, groups of 16 larvae at their final instar stage (body weight, 400 ± 20 mg) were inoculated (10 μl/larva) directly in the haemocoel, via the last left proleg, with the selected peptides (12.5 mg/kg). Control groups consisted of larvae untouched or inoculated with 10 μl of saline solution. Larvae were then transferred to clean Petri dishes (one for each experimental group), incubated at 37 °C in the dark for 9 days, and scored daily for survival.

To evaluate potential therapeutic activity of selected peptides, larvae (16/group) were inoculated with 10 μl of a *C. albicans* SC5314 suspension (5 × 10^5^ cells/larva) via the last left proleg. 60 min later, larvae were injected via the last right proleg (single injection of 10 μl) with the selected peptides (12.5 mg/kg) or saline (control). Larvae injected with saline solution alone served as a further control. Larval survival was monitored daily for up to 9 days after infection. Survival curves were compared by the log rank (Mantel-Cox) test using Graph Pad Prism software. A *p* value < 0.05 was considered significant.

### Evaluation of apoptosis profile in *C. albicans* after treatment with selected peptides

Peptide-induced apoptosis in *C. albicans* SC5314 cells was evaluated by the Muse cell analyzer (Merck Millipore, Germany) using the Muse annexin V and dead cell assay kit, as previously described^30^. Briefly, yeast cells were suspended in 100 μl water (5 × 10^5^ cells/ml) in the absence (control) or presence of the selected peptides at a concentration equal to 2 × EC_50_ and incubated for 30 and 60 min at room temperature. Treated and control cell suspensions (90 μl) were added to 10 μl of 10% bovine serum albumin and 100 μl of Muse kit reagent 20 min before the measurements, maintaining the mixture in the dark. Data were acquired according to the manufacturer’s instructions. The candidacidal activity of the peptides under the adopted conditions was verified by CFU assay.

### Confocal microscopy studies

Confocal microscopy studies were performed on living yeast cells according to a procedure previously described with minor modifications^31^. Briefly, 4 × 10^5^ *C. albicans* SC5314 cells, grown overnight at 30 °C with shaking (100 rpm) in yeast extract, peptone, and dextrose broth, were seeded on coverslips mounted in a special flow chamber (20 μl). After 30 min, FITC-labeled L12P and L18R peptides were added (final concentration 200 μg/ml). Propidium iodide was added (1.5 μM) at predetermined times. The candidacidal activity of the peptides, under the adopted conditions, was verified as described above.

### Transmission and scanning electron microscopy studies

For transmission electron microscopy studies, *C. albicans* SC5314 germinating cells were obtained by inoculating 1 ml of the yeast broth culture (see above) in 10 ml of medium 199, then incubated for 90 min at 37 °C with shaking (150 rpm). Candidal suspensions (approximately 10^7^ cells) containing equal numbers of budding and germinating cells were incubated for 60 min in the absence (control) or presence of the selected peptides (125 μg/ml) in a final volume of 50 μl. After incubation, cells were pre-fixed for 5 min with 5% glutaraldehyde in 0.1 M phosphate buffer, pH 6. Pellets obtained after centrifugation were packed in solidified 3% agarose. Yeast cells in agarose blocks were fixed for 3 h at room temperature with 2.5% glutaraldehyde in phosphate buffer, then kept overnight at 4 °C. Post-fixation was performed with 1% osmium tetroxide for 30 min, followed by dehydration with acetone gradient (25–100%). A prolonged infiltration protocol using multiple changes of Durcupan ACM epoxy resin was adopted^32^, followed by embedding and hardening of the resin for 72 h at 58 °C. Semi-thin sections (0.75 μm) were stained with methylene blue and safranin for observation in an optical microscope to confirm the presence of an adequate amount of *Candida* cells. Ultra-thin sections (80 nm) contrasted with 4% uranyl acetate and Reynolds’ lead citrate were examined in a Philips EM 208S transmission electron microscope (Fei Europe, Eindhoven, The Netherlands).

For scanning electron microscopy studies, approximately 4 × 10^5^ cells, prepared as above, were incubated with the selected peptides (125 μg/ml) for 60 min in a final volume of 20 μl. Slides for microscopic examination were prepared as previously described^30^. Briefly, slides were fixed with a glutaraldehyde-sodium cacodylate buffer, washed in sodium cacodylate, then dehydrated by immersion in alcohol solutions in a 25 to 100% gradient. After washing in acetone, samples were dried in liquid CO_2_, fixed on a support and gold coated in an ion-sputtering unit. The samples were observed in a Philips 501 scanning electron microscope (15 kV).

The candidacidal activity of the peptides under the adopted conditions was verified as described above.

### Data Availability

The data generated or analysed during this study are included in this published article or available from the corresponding author.

## Electronic supplementary material

Supplementary Information
