# Supplementary material for: Fungicidal activity of peptides encoded by immunoglobulin genes
Source: Sci Rep. 2017 Sep 7;7:10896. doi: 10.1038/s41598-017-11396-6 (PMC5589769; doi:10.1038/s41598-017-11396-6)
Supplement: Supplementary file 1 — Supplementary Information [file 41598_2017_11396_MOESM1_ESM.pdf]

Fungicidal activity of peptides encoded by immunoglobulin genes

Luciano Polonelli[1], Tecla Ciociola[1], Martina Sperindè[1], Laura Giovati[1], Tiziana D'Adda[1], Serena Galati[2], Luiz R. Travassos[3], Walter Magliani[1], Stefania Conti[1]*

[1] Department of Medicine and Surgery, University of Parma, Parma, Italy; [2] Department of Chemistry, Life Sciences and Environmental Sustainability, University of Parma, Parma, Italy; [3] Experimental Oncology Unit, Federal University of São Paulo (UNIFESP), São Paulo, Brazil.

*Corresponding author

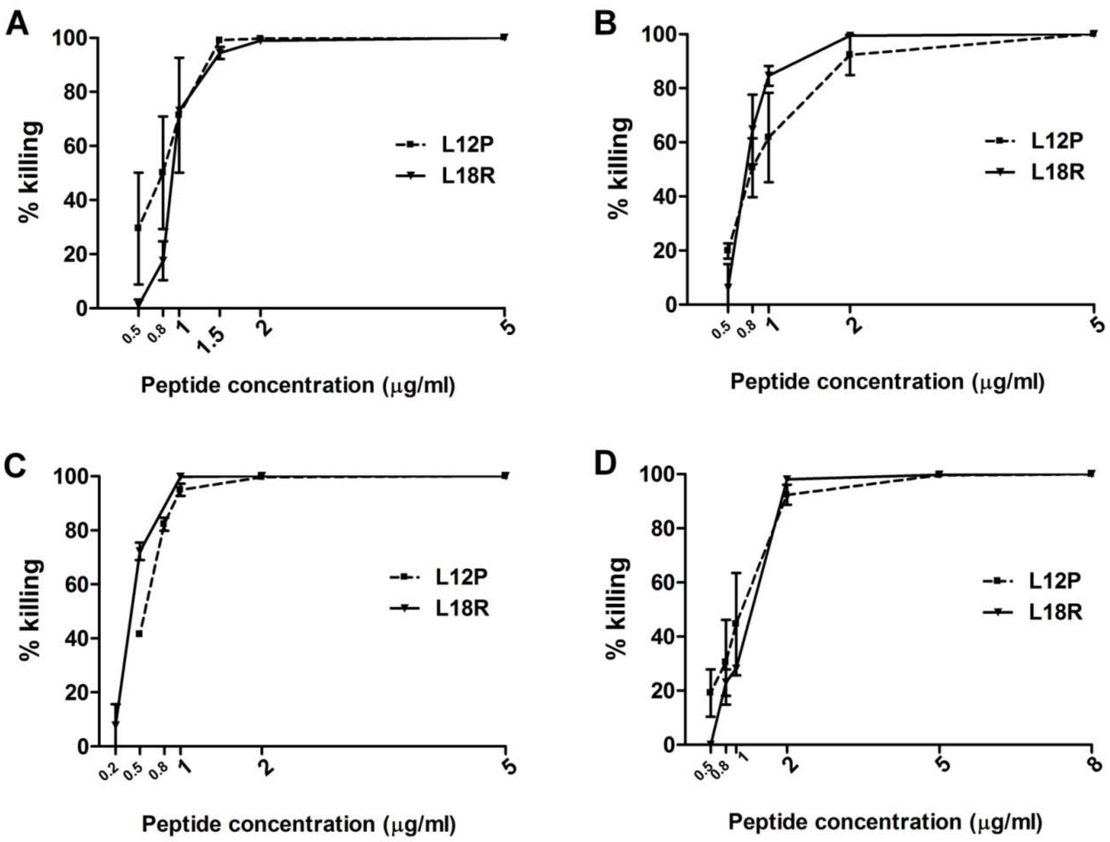

Supplementary Figure S1. Fungicidal activity *in vitro* of L12P and L18R against yeast strains of different species. Panel A: *Candida albicans* SC5314; Panel B: *C. glabrata* OMNI32; Panel C: *Cryptococcus neoformans* 6995; Panel D: *Malassezia furfur* 101. Viable yeast cells were incubated in the absence (control) or presence of peptides at different concentrations. After 6 hours the yeast suspensions were plated on Sabouraud dextrose agar, and colony forming units were enumerated after 48 h. The activity is expressed as percent killing with reference to the number of colonies in controls; data represent mean values (± standard deviation) from independent experiments performed in triplicate.

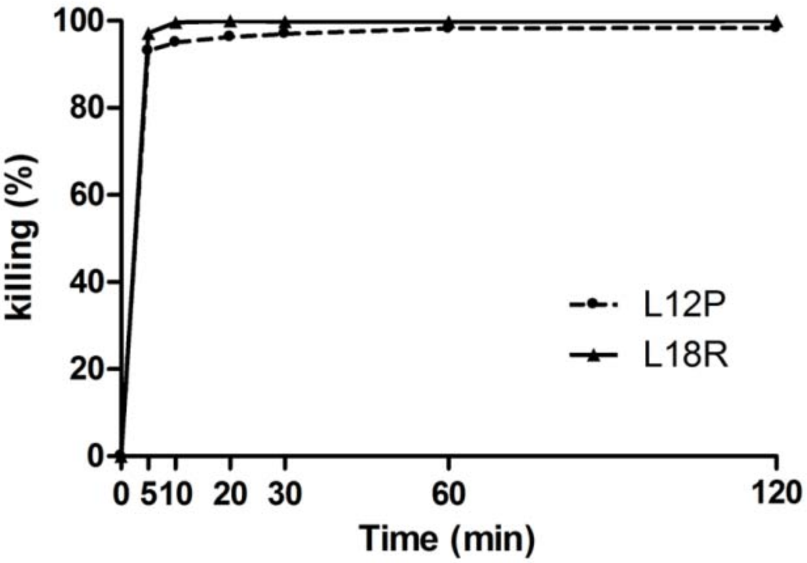

Supplementary Figure S2. Time kinetics up to 120 minutes of *in vitro* L12P and L18R killing of *Candida albicans* SC5314 cells. Viable yeast cells were incubated in the absence (control) or presence of peptides at their minimal fungicidal concentration (5 µg/ml). At different times the yeast suspensions were plated on Sabouraud dextrose agar, and colony forming units were enumerated after 48 h. The activity is expressed as percent killing with reference to the number of colonies in controls; data represent mean values from independent experiments (variability ≤10%) performed in triplicate.

Supplementary Table S1. ***In vitro*** **haemolytic activity of the investigated peptides.**

Peptide	Haemolysis (%)*							
	50 µM		100 µM		250 µM		500 µM	
	30 min	120 min	30 min	120 min	30 min	120 min	30 min	120 min
**L12P**	0.32	0.00	0.16	0.00	0.00	0.00	0.00	0.00
**W12K**	0.00	0.00	0.00	0.00	0.21	0.00	0.16	0.00
**G10S**	0.00	0.59	0.00	0.00	0.11	0.51	0.53	0.98
**L18R**	0.00	4.40	0.00	5.80	0.00	7.54	0.20	9.66

* values of haemolysis were obtained after 30 and 120 minutes of incubation of erythrocytes with the peptides at different concentrations, in comparison to erythrocytes suspended in 1% Triton X-100 (100% haemolysis) and PBS (0% haemolysis), by measuring supernatant absorbance at 540 nm.

Supplementary Table S2. ***In vitro*** **cytotoxic activity of the investigated peptides against LLC-MK2 cells.**

	**Cell viability (%)***			
**Peptide**	**50 µM**	**100 µM**	**250 µM**	**500 µM**
**L12P**	97.96	96.95	100.00	100.00
**W12K**	100.00	98.76	100.00	100.00
**G10S**	100.00	100.00	98.18	100.00
**L18R**	99.05	100.00	90.14**	83.88**

* cell viability values are expressed as percent ratio T/C, where T represents the mean value of fluorescence intensity for cells treated with the peptides at different concentrations and C the mean value of control cells (in the absence of peptides) obtained after 24 h of incubation in a viability assay using resazurin as indicator.

** statistically significant difference in mean values of fluorescence intensity versus untreated cells (p<0.01).

Supplementary Table S3. Genotoxic activity of the selected peptides against peripheral blood mononuclear cells evaluated by alkaline Comet assay.

	Tail Intensity (%)*			Visual score**		
**Peptide**	**0 µM**	**5 µM**	**10 µM**	**0 µM**	**5 µM**	**10 µM**
**L12P**	0.37 ± 0.04	0.55 ± 0.08	0.21 ± 0.14	104 ± 2.83	109 ± 4.24	103 ± 1.41
**L18R**	0.37 ± 0.04	0.22 ± 0.02	0.21 ± 0.06	104 ± 2.83	106 ± 1.00	103 ± 1.41

* Percentage of DNA in comet tail (median values ± standard deviation).

** Calculated after attribution of the observed cells to a class (0 to 4) on the basis of the length of the comet tail, according to the formula: number (no.) of class 0 cells + 2 × (no. of class 1 cells) + 3 × (no. of class 2 cells) + 4 × (no. of class 3 cells) + 5 × (no. of class 4 cells).
